# The Impact of Cultural Memory and Cultural Identity in the Brand Value of Agricultural Heritage: A Moderated Mediation Model

**DOI:** 10.3390/bs13020079

**Published:** 2023-01-17

**Authors:** Qionge Zheng, Sunbowen Zhang, Jingxuan Liang, Youcheng Chen, Weijiao Ye

**Affiliations:** 1 Anxi College of Tea Science, Fujian Agriculture and Forestry University, Quanzhou 350002, China; 2 College of Humanities & Social Development, Nanjing Agricultural University, Nanjing 210095, China; 3 College of Business Administration, Capital University of Economics and Business, Beijing 100070, China

**Keywords:** agricultural heritage, cultural memory, cultural identity, brand value, Anxi Tieguanyin Tea Culture System

## Abstract

Improving the brand value of agricultural cultural heritage can promote the development of the local social economy. Meanwhile, cultural memory and brand value are inseparable. Therefore, this study took the Anxi Tieguanyin Tea Culture System as the research object, collected 679 questionnaires, adopted a structural equation model, and applied SPSS 26.0 and Amos 24.0 software (IBM; Armonk, NY, USA) to study the influence of agricultural heritage cultural memory on brand value. This study innovatively develops a scale of agricultural heritage cultural memory, enriches the outer edge of cultural memory theory research, broadens the vision of agricultural heritage research, and provides a useful reference for the inheritance of agricultural heritage and the promotion of brand value.

## 1. Introduction

Agricultural heritage inherits the foundation of rural culture and has profound historical and cultural heritage. In 2002, the Food and Agriculture Organization of the United Nations (FAO) launched the “Globally Important Agricultural Heritage Systems (GIAHS)” protection project [1], emphasizing the multiple roles and values of agricultural heritage [2], aiming to protect the world agricultural heritage system. Initially, it mainly focused on the cases of developing countries [3].

Agricultural heritage has cultural attributes and historical inheritance, and history is the retention of memory. The inheritance of agricultural heritage by a group constitutes cultural memory. Culture is passed down from generation to generation by the group through rituals, festivals, symbols, words, and other carriers to form cultural memory [4]. It is the inheritance of symbolic experience and knowledge and continues the life of history and culture [5]. Culture and history play a constitutive role in human psychology and have an important impact on conscious and unconscious human experience [6]. This study believes that introducing the concept of cultural memory into agricultural heritage can systematically analyze the content of agricultural heritage from the perspective of the group.

The group has a common memory of culture and forms a consistent cultural identity to the concept of culture in memory through media communication. Cultural identity is a “common personality” developed, activated, and modified by social actors in the context of social and historical interaction according to the specific problems that lead them to take action [7]. Cultural identity will enhance the group’s preference and purchase of brands [8]. Agricultural heritage derives many product brands of agricultural heritage, and brand assignment drives the economic and social development of the heritage site [9]. The direct effect of products is the group, so the direct embodiment of brand value is the loyalty of the group to the brand [10]. The cultural memory of agricultural heritage is inherited by the group, and the brand value depends on group recognition.

Although culture and brand value belong to “culture, value system, and social organization”, which is one of the five criteria for evaluating GIAHS, there are few studies that directly discuss its culture and brand value [3]. Therefore, this study aims to explore the role of culture in the brand development of agricultural heritage and enriches the research dimension of agricultural heritage. With the rapid development of Internet technology, the use of social media regulates the impact of network diversity on cultural knowledge [11]. The emergence of social media makes the communication of group cultural memory more rapid and extensive, meaning the fermentation speed of group cultural identity in social media is faster and faster, and the impact on brand value is also deepened. Social media plays an important role in cultural memory inheritance and brand value. However, the existing research has not yet discussed the impact of social media interaction on agricultural heritage cultural memory and brand value. As a result, we can infer that in the context of social media, the cultural memory of agricultural heritage is widely disseminated, which promotes the cultural identity of the group and further enhances the brand value. Therefore, based on the group perspective in the social media environment, this study found an interesting research gap: whether the cultural memory of agricultural heritage has an impact on its brand value, and what role social media interaction and group cultural identity play in it.

Based on this, this study selects the Anxi Tieguanyin Tea Culture System as a typical case, according to the distinctive characteristics of agricultural heritage with profound historical brand and cultural attributes, introducing the concept of cultural memory, adopting a structural equation model, and using SPSS 26.0 and AMOS 24.0 (IBM; Armonk, NY, USA) to analyze the relationship between the cultural memory and brand value of agricultural heritage. This paper aims to provide a new perspective for academic circles and managers, and also provide suggestions for the development of agricultural heritage from the perspective of whole process management.

## 2. Literature Review and Hypotheses Development

### 2.1. Agricultural Heritage

The selection criteria of cultural heritage have changed: while initially, the historic and artistic values were the only parameters, other additional ones have now been added: the cultural value, its value of identity, and the capacity of the object to interact with memory [12]. In the field of agriculture, the Food and Agriculture Organization of the United Nations (FAO) has proposed “agricultural heritage”. This concept has been widely recognized since the FAO launched the Globally Important Agricultural Heritage Project. In this view, agricultural heritage refers to a unique land use system and agricultural landscape with rich biodiversity that can simultaneously meet the needs of local social, economic, and cultural development under the long-term co-evolution and dynamic adaptation of villages and their environment [13]. Some scholars believe that the definition given by the GIAHS is a narrow concept, and the broad agricultural heritage should integrate the material and intangible relics closely related to human agricultural activities in different periods [14], namely agricultural heritage, to include the agricultural production experience and agricultural life experience in the narrow concept [15]. Agricultural heritage in a broad sense generally includes 10 aspects, such as agricultural sites, agricultural species, agricultural engineering, agricultural landscape, agricultural settlements, agricultural techniques, agricultural documents, agricultural specialties, and agricultural folk culture [16]. In conclusion, according to the content and direction of this research, the broad agricultural heritage is chosen as the basic concept.

### 2.2. Cultural Memory

Cultural memory is the sum of rituals, festivals, symbols, characters, etc. that are unique and reusable in a specific society or era [4]. The research on memory originated from the perspective of psychology. Memory is regarded as an individual spiritual existence, but Bartlett believes that memory should be regarded as a constructive process under social conditions [17]. Starting from the social foundation of memory, Assmann pointed out the cultural foundation of memory and put forward the theory of cultural memory for the first time [4]. Assmann believed that cultural memory was preserved by memory media and inherited through the display of intellectuals such as priests, teachers, artists, and officials. Cultural memory needs to continue its life with the help of the space–time dimension, and the three elements of time, space, and group are indispensable. Assmann explains the law of civilization development and cultural memory from the perspective of cultural inheritance. Assmann argues that memory is controlled by a “society of symbols and symbols” that is part of a cultural system [4].

Cultural memory is related to brand personality and performance and has a significant impact on brand performance [18]. Brand culture is an important attribute to promote the development of brand value and an important embodiment of brand core value [19]. In the study of a cultural city as one of the urban brands, it is verified that the cultural characteristics of the city have a positive impact on the promotion of urban brand value [20]. Many brands in China use cultural heritage in their brand strategy and bring cultural heritage into brand cultural elements to build brand value. China’s rich cultural heritage has positive significance for their brand competition [21].

The cultural and heritage industry provides a sense of belonging and identity through links to time and place experiences [22]. An important aspect of cultural identity is the common history and memory, which can be presented through heritage, which is a trace of the past that society chooses to protect [22]. As far as cultural location is concerned, tourists’ cultural contact directly and indirectly affects their revisiting intention through cultural memory. Cultural memory dominates tourists’ behavior decision-making, and the depth of cultural memory is an important part of perceiving local culture and forming cultural identity [23]. Based on the above theory and literature analysis, this study proposes the following research hypothesis:

**Hypothesis** **(H1).** *Cultural memory has a significant positive impact on brand value.*

**Hypothesis** **(H2).** *Cultural memory has a significant positive impact on cultural identity.*

### 2.3. Cultural Identity

Cultural identity is the specific expression of social identity in the cultural context [24]. Collective identity is the de-self-association based on group member relations, and culture is the cognitive element of social identity [25]. Therefore, cultural identity is the core concept of collective identity. Universal identity belief and consumer-based identity belief constitute consumer cultural identity [26]. Social actors develop, activate, and modify their “common personality” in the context of social and historical interaction according to the specific problems that lead them to take action to form cultural identity [7].

Cultural brand embodies the cultural identity of a group, including the identity of myths and cultural roots, as well as the consistency of tracing back to the past and longing for the future [27]. This study extends the research of brand to the field of culture and history, which shows that the concept of culture and history exists in the producer’s intention or product concept, and also exists in the individual consumer or brand value [28]. Cultural identity improves consumers’ preference and purchasing power for local brands [8].

Because the delay of history denies a single objective truth, the group weaves various fragments of the “past” special moment together through “cultural memory”. After this fragment is recognized by the group, it is reinterpreted to form cultural identity and expressed through various media [29]. Memory is not an unchanging legacy, but a plastic resource for sharing stories about the past from the perspective of the future and the present and an exchange behavior for transmitting past knowledge [30,31]. Historical souvenirs or artworks combine various cultural symbol systems to form a memory system, which is a symbolic space for memory and forgetting, in which the time sequence of the past and the present is constantly recombined [32]. Cultural memory promotes the subject to produce the consciousness of belonging to a group, and then produce cultural identity. The group have a sense of identity with the same culture, which promotes them to have a sense of belonging to the brand containing this cultural memory, thus enhancing the brand value. For this reason, this study proposes the following hypothesis:

**Hypothesis** **(H3).** *Cultural identity has a significant positive impact on brand value.*

**Hypothesis** **(H4).** *Cultural identity plays a mediating role in the relationship between cultural memory and brand value.*

### 2.4. Brand Value

Brand value comes from the general human value system proposed by Schwartz and his colleagues [33]. One way for brands to carry cultural meaning is to instill human values into brands. According to this point of view, brand value can be regarded as an abstract brand concept, which represents the brand concept of human value [34], thus making the brand play the functional value of human beings [35]. Kucharska et al. regards the definition of contemporary brand value as the strategic result of the company’s marketing initiatives [36]. The brand value provided by manufacturers provides emotional value for consumers and reasonable value for their commercial customers. The operational efficiency of brand value is reflected as an important element of providing value to consumers and commercial customers [37]. In order to attract consumers’ attention to the brand and realize cultural resonance, brand advertisers increasingly talk about diversified words through social media, which helps to solve the problem of the dynamic, tense, and dialectical relationship between consumers and brands [38]. Almeida et al. pointed out that different types of territorial brands coexist in society, generated by different power relations, whose interests in their creation and management also differ [39].

### 2.5. Social Media Interaction

Social media interaction refers to the whole process of sending and receiving information, including four parts: information source, information transmission channel, receiver, and feedback. Information affects the perception and action of groups through symbolic communication, and social media ensures the realization of two-way instant interactive communication. Groups re-integrate the received information with the help of media, which further affects their original cognitive thinking [40].

The development of technological society has spawned cross regional networks, making it possible to realize the globalization of diversified cultural memory [41]. Social media-driven debates on memory knowledge transcend history itself and show the complex cultural debates of the contemporary era [42]. In network society, information-based cultural heritage has become the source of new cultural symbols, which exist through the continuous regeneration and dissemination of symbols. The combination of symbols and regenerative symbols constitutes information-based cultural heritage. Therefore, the group’s understanding of cultural heritage is in a state of constant change [43]. With the rapid development of Internet technology, social media has become an important channel for the group to produce cultural identity. The operation logic of social media and mass media is quite different, which leads to different ways of producing content, distributing information and using media, thus affecting groups’ understanding of cultural knowledge [44].

Cultural identity is not only an integral part of the relationship between media and audience, but also an element of the relationship between media and audience [45]. Cultural intermediaries use social media to create real and unique experiences between brands and consumers [46]. Social media is very important in promoting the integration of the group into the new culture [47]. Chen et al. found that social media has an important influence on women’s cultural identity in the study of the role of social media on immigrants’ cultural identity [48]. Consumers and organizations communicate effectively beyond the limitation of time and space through social media, which is an important means of cultural change and the driving force of consumption choice. The company encourages consumers to experience more value through the participation of social media, creates a multicultural atmosphere, and promotes cultural exchanges [49]. Consumers’ participation in social media affects their willingness to adopt cultural products [47].

The brand community established on social media has a positive impact on community logo and value-creation practice [50]. The work of social media influencers on the Internet makes a significant contribution to the generation of brand value. The work of social media influencers is a form of brand value creation [51]. The use of social media has a positive impact on the embodiment of brand value. Consumers connect human characteristics with brands through social media, which makes them have a highly anthropomorphic perception of brands [52]. Consumers tend to interact and participate in the discussion of such products through social media, so as to increase their interest in products and enhance their willingness to consume [53]. The overall brand value depends on the subjective evaluation of customers, and social media interaction is the key link in the formation of subjective evaluation [54]. The degree of group use of social media is positively correlated with the improvement of brand value [55].

With the continuous development of Internet technology, the phenomenon of social media interaction is becoming increasingly prominent. Cultural memory is widely spread through social media. The Internet has become the main channel for the wide-ranging and efficient dissemination of cultural memory. As a communication channel, social media has an important impact on the communication effect. In the context of the Internet, the information interaction between individuals is enhanced, so as to strengthen the individual’ s impression of cultural memory, and then deepen the cultural identity, and ultimately enhance the brand value. This study therefore proposed the following hypotheses based on the foregoing research:

**Hypothesis** **(H5).** *Social media interaction regulates the relationship between cultural memory and brand value.*

**Hypothesis** **(H6).** *Social media interaction regulates the relationship between cultural memory and cultural identity.*

**Hypothesis** **(H7).** *Social media interaction regulates the relationship between cultural identity and brand value.*

**Figure 1:**
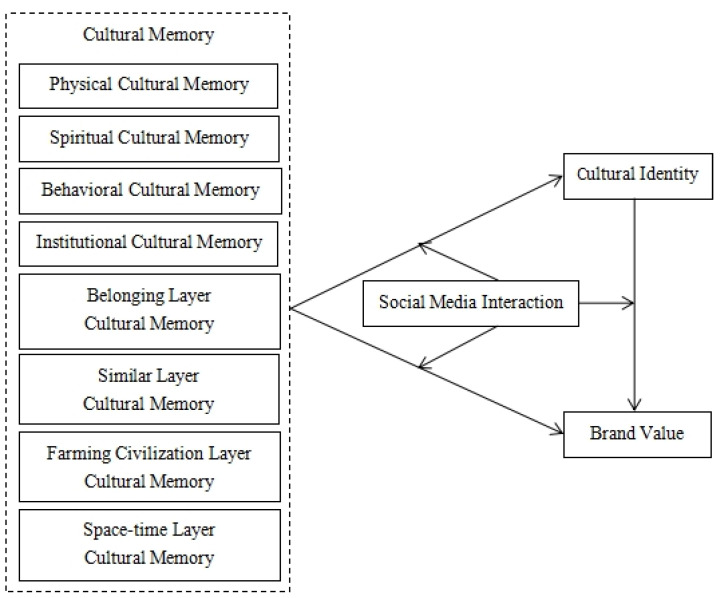
The theoretical model of this study.

To sum up, agricultural heritage is an important concept newly proposed in recent years. At present, scholars’ research on the brand value of agricultural heritage is still in the blank stage. Therefore, against the background of the rapid development of the Internet, according to the unique cultural attributes of agricultural heritage brands, it is of great theoretical contribution and practical significance to explore the influence of cultural memory of agricultural heritage on brand value under the social media environment. Based on the above research hypothesis, this study constructs a theoretical framework, as shown in Figure 1.

## 3. Research Methods

### 3.1. Research Object

In this study, the agricultural heritage of Anxi Tieguanyin Tea Culture System was selected as a case study. Anxi Tieguanyin Tea Culture System became the second batch of China Nationally Important Agricultural Heritage Systems (China-NIAHS) in 2014 [56]. Since then, Anxi County has strengthened the protection, development, inheritance, and dissemination of Anxi Tieguanyin Tea Culture System, and achieved positive results [56]. It was officially recognized as a Globally Important Agricultural Heritage System (GIAHS) in 2022. Anxi Tieguanyin Tea Culture System originated in the late Tang Dynasty and flourished in the Ming and Qing Dynasties, with a long historical and cultural heritage. It was selected for the list of mutual recognition and mutual protection products of “China Europe 100 + 100” geographical indication products and the “national brand project”. For four consecutive years, it ranked first in the regional brand value of Chinese tea, won the gold award of Chinese famous tea at the Centennial World Expo, the most familiar and favorite brand of Chinese agricultural products by foreign businessmen, and became the representative symbol of Chinese tea in the world with high brand recognition. Based on the extensive representativeness of agricultural heritage of Anxi Tieguanyin Tea Culture System, this study selects it as a research case to the influence mechanism of cultural memory of agricultural heritage on its brand value.

### 3.2. Measurement

The measurement of each structure in this study was carried out in the form of Likert level 7 scale, and the answers ranged from 1 point (extremely inconsistent) to 7 points (extremely consistent). This study involves four dimensions, among which cultural identity, brand value, and social media interaction all have maturity scales, but there is no maturity scale for cultural memory at present. Therefore, in this study, the scale of cultural memory is coded by grounded theory on the network texts related to agricultural heritage, and the extracted results are verified by Delphi method. The reliability and validity of the questionnaire were tested and the model was modified, and the final scale of cultural memory of agricultural heritage was obtained. The data test results during the development of the cultural memory scale are as follows.

First of all, in order to avoid the problem of homology deviation, according to the suggestions of Podsakoff [57] and Ramkissoon [58], HS Chen [59] single factor is used to test the common method bias, so as to test the influence of a single sample source on the increase or decrease in correlation between dimensions. Using unrotated principal component analysis, 8 factors with eigenvalue greater than 1 were extracted. Their cumulative explained variance to total variance is 71.79%, and within which the cumulative explained variance of the first principal components is 17.95%. Therefore, there was no serious common method bias in the data of this study.

The reliability and validity of the cultural memory scale are Factor Loading > 0.6, Cronbach’s α > 0.7, Composite Reliability value > 0.7, Average Variance Extracted value > 0.5. In order to simplify the model and reduce the estimated parameters of the structural equation model, a second-order model is constructed for cultural memory; that is, the first order is eight factors, the second-order is cultural memory, and its fitting index is: χ^2^/df = 3.068, GFI = 0.855 AGFI = 0.835, CFI = 0.905, RMSEA = 0.06, which does not meet the recommended value range (χ^2^/df < 3, GFI > 0.9, AGFI > 0.9, CFI > 0.9, RMSEA < 0.08). Hence, model modification is required. The chi-square ratio between the first-order eight-factor correlation model and the second-order factor is 98%; that is, the target coefficient = 0.98, and the closer the target coefficient is to 1, the better it is [60,61]. Therefore, the second-order model in this study is acceptable.

By deleting the items with larger chi-square value, the model is modified, and the final items of the scale are obtained by testing the fitting degree of the modified model. Finally, we delete the items “I know Anxi Tieguanyin Tea tree”, “I know Anxi Tieguanyin Tea mountain system”, “I know Anxi Tieguanyin Tea art”, “I know Anxi Tieguanyin Tea brewing method”, “I know Anxi Tieguanyin Tea making technology”, “I know Anxi Tieguanyin Tea planting technology”, “I know Anxi Tieguanyin is applying for the Globally Important Agricultural Heritage Systems (GIAHS)”, “I have heard that Anxi Tieguanyin Tea has become a national ceremony tea many times”. The fitting index of the modified second-order factor model is: χ^2^/df = 2.151, GFI = 0.922, AGFI = 0.905, CFI = 0.954, RMSEA = 0.045, which all meet the recommended value range. In addition, the chi-square value between the first-order nine-factor related model and the second-order factor is 96%; that is, the target coefficient is 0.96. Therefore, the second-order model is acceptable, the simplified scale items are applicable, and the final items of cultural memory of agricultural heritage are obtained.

The maturity scales of the other three dimensions are cultural identity, including 6 items, mainly based on the research of Peng [62]; brand value, including 5 items, borrowed from the research of Ghosh [63] and Kim [64]; social media interaction, including five topics, which are based on the research of Albert [40]. The specific items of the scale are shown in Table 1.

### 3.3. Data Collection

The research object of this study was divided into two parts: one is the group in the heritage site, the other is the group outside the heritage site. The research time in the heritage site was from 2 August to 9 August 2021. Then, the members of the research team took out research samples outside the heritage site. The research time was from 12 August to 25 August 2021. The research objects in the heritage site were mainly Anxi Tieguanyin Tea planters, tea enterprise practitioners, teachers and students of Anxi College of Tea Science of Fujian agriculture and Forestry University and Anxi Tea Vocational and Technical School, government staff of Anxi County Agriculture Bureau, Anxi newspaper, Anxi TV station, and official media practitioners of “talking about tea”. The research objects outside the heritage site were mainly enterprise practitioners, teachers and students at colleges and universities, government staff of relevant agricultural departments, and practitioners of tea related We-Media in various places. For the investigation of growers, considering that some local tea farmers have not learned Mandarin, we asked local students to do accompanying translation in Minnan, communicate with tea farmers, and fill out the questionnaire to ensure the validity of the questionnaire. For the research of the government, official media and universities, we applied for a research letter and entered the workplace of the unit to conduct standardized research to ensure the credibility of the questionnaire. For the investigation of We-Media practitioners, we found the account number related to Anxi Tieguanyin Tea by inquiring the WeChat official account and Short Videos of local services in various places, communicated with the information publisher team by private letter in the background, and met offline to fill out the questionnaire. In the end, 679 questionnaires were collected, and 582 valid data were obtained after eliminating invalid questionnaires, with an effective rate of 85.7%, the covariance matrix of data is shown in Table S1. Demographic profile of the sample is provided in Table 2.

## 4. Results

### 4.1. Common Methods Bias Test

The data collected in this study come from different times and places. However, cultural memory, cultural identity, brand value, and social media interaction from the same source are adopted. In order to avoid the problem of homology deviation, according to the suggestions of Podsakoff et al. and Ramkissoon et al., Harman single factor is used to test the common method bias, so as to test the influence of a single sample source on the increase or decrease in correlation between dimensions [57]. Using unrotated principal component analysis, 11 factors with an eigenvalue greater than 1 were extracted. Their cumulative explained variance to total variance is 72.55%, and within which the cumulative explained variance of the first principal components is 13.44%. Therefore, there was no serious common method bias in the data of this study.

### 4.2. Exploratory Factor Analysis

Since cultural memory in this study is based on grounded theory, its reliability remains to be verified. Therefore, in order to ensure the scientificity of this study, and to ensure that the scale used in this study has sufficient reliability and validity, this study conducts exploratory factor analysis on the pre-survey samples based on principal component analysis. The results of exploratory factor analysis in Table 3 show that 11 common factors are generated after principal component analysis, and the eigenvalues of the common factors after aggregation are all greater than 1, and the load on each variable factor exceeds 0.6 from the factor load after rotation of each measurement item. Therefore, this research scale is more suitable for further empirical analysis.

### 4.3. Reliability and Validity Test

This study uses confirmatory factor analysis to analyze the reliability and validity of data by Anderson [65]. As can be seen from Table 4, the Factor Loading of each item is >0.6, so the measurement index variables of each dimension can effectively reflect the potential characteristics of the corresponding dimensions. The Cronbach’s α of all dimensions are greater than 0.7, which indicates that the sample data have good reliability. The Composite Reliability value of each dimension is >0.7, indicating that the measurement questions of each potential variable are internally consistent. The Average Variance Extracted value of each dimension is >0.5, indicating that each dimension of the measurement model has good convergence validity. From the results of Table 5, the square root of each dimension AVE is higher than correlations among the corresponding latent variables, providing evidence of discriminant validity.

### 4.4. Model Fitting Test and Correction

Model fit refers to the consistency between the theoretical model and the sample model. In view of the room for improvement in χ^2^/df, AGFI and CFI indices of the hypothetical model, the hypothetical model should be further modified. This study modifies the model by deleting the items with large chi-square value. We delete items CI5 and BV1 to obtain a modified model, and the fitting index results are shown in Table 6. All the matching indexes of the modified model meet the requirements and are better than those of the initial model.

### 4.5. Research Hypothesis Test

Firstly, taking cultural memory as the independent variable and brand value as the dependent variable, a direct effect model is established after controlling personal information such as gender, age, education, and income. From the results of Table 7, the results of Equation (1) show that there is a significant correlation between cultural memory and brand value (β = 0.254, *t* = 3.258, *p* < 0.01). Furthermore, according to the suggestion of experiment of Williams and Vaske, we choose to report the confidence interval of Bias-corrected [66]. The non-parametric Bootstrap method is used to estimate the parameters of 2000 random samples. The 95% confidence interval is [0.089, 0.415], which does not include 0. Therefore, H1 is supported.

Then, on the basis of the original model, cultural identity is added as a mediator variable to test the significance of the mediating effect. We use the mediating effect model 4 of the Process plug-in in SPSS software [67]. After controlling personal information such as gender, age, education, and income, we obtain the test results of mediating effect as shown in Equations (2) and (3) (Table 7). The results show that the influence of cultural memory on cultural identity (β = 0.425, *t* = 5. 263, *p* < 0.01) and cultural identity on brand value (β = 0.094, *t* = 2. 348, *p* < 0.01) are both significant. Therefore, Hypothesis H2 and H3 are verified.

Cultural identity plays a partial mediation role in the impact of cultural memory on brand value, and Table 8 shows the value of the mediating effect. In all, 84.3% of the effects of cultural memory on brand value are direct effects, and 15.7% is transmitted through the mediator variable cultural identity. Hence, Hypothesis H4 is supported.

Afterwards, we validate the moderating effect of social media interaction based on the mediation model. The mediating effect model 59 of the Process plug-in in SPSS software is used to test the adjusted mediation model under the control of gender, age, education, and income [67]. The results are shown in Table 9. It can be seen from Equations (4) and (5) that after putting social media interaction into the model, the product of cultural memory and social media interaction has no significant impact on brand value and cultural identity (brand value: β = −0.026, *t* = −0.431, *p* > 0.05; cultural identity: β = −0.055, *t* = −0.959, *p* > 0.05), the product of cultural identity and social media interaction has a significant impact on brand value (β = 0.079, *t* = 2.410, *p* < 0.05). It can be seen that social media interaction is not enough to regulate the direct impact of cultural memory on brand value or the impact of cultural memory on cultural identity; that is, H5 and H6 are not supported; however, social media interaction can moderate the influence of cultural identity on brand value. Hence, Hypothesis H7 is verified.

**Figure 2:**
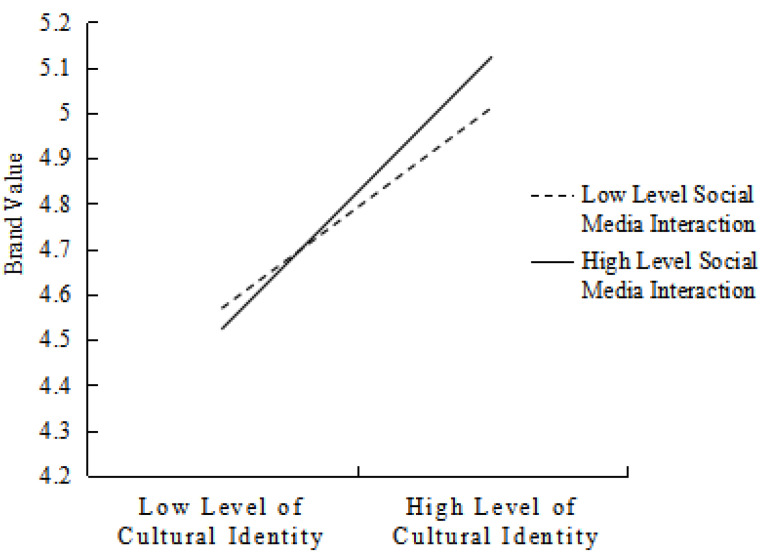
The moderating effect of different social media interaction levels on the relationship between cultural identity and brand value.

Furthermore, in order to present the adjustment effect more intuitively, a slope diagram of the adjustment effect of one standard deviation above and below the average value is drawn. A simple slope analysis of the moderating effect of social media interaction in the influence of cultural identity on brand value is shown in Figure 2.

From the results in Table 10, it can be seen that when the score of social media interaction is average minus one standard deviation; that is, the level of social media interaction is low, the indirect effect of cultural memory on brand value through cultural identity is not significant (Boot 95% CI cross 0); when the score of social media interaction is the average plus a standard deviation, that is, the level of social media interaction is high, the indirect effect of cultural memory on brand value through cultural identity is significant (Effect = 0.067, Boot95% CI does not cross 0). The mediating effect of social media interaction is 0.040, and Boot95% CI is [0.004, 0.081], which does not cross 0, so there is a moderated mediator effect. In conclusion, at the medium and high level of social media interaction, the mediating effect of cultural identity on the relationship between cultural memory and brand value is significantly moderated by social media interaction, and there is a moderated mediator effect; that is, Hypothesis H7 is supported.

**Figure 3:**
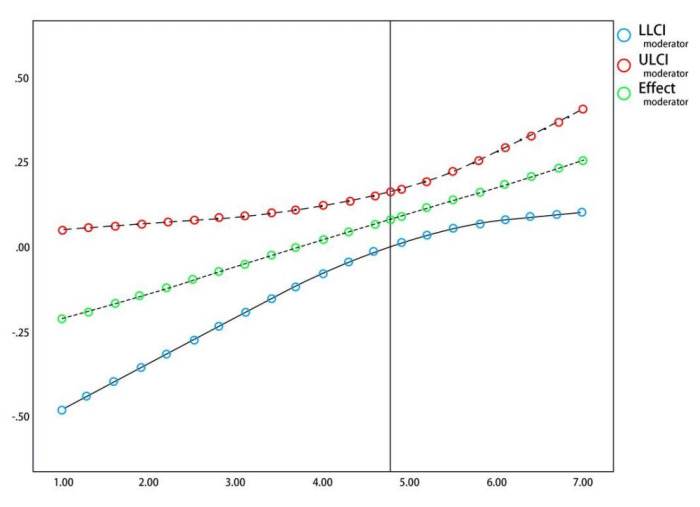
The moderating interval of social media interaction on the relationship between cultural identity and brand value.

Finally, in order to further quantitatively test the moderating effect, the Jonson-Neyman technique is used to detect the moderating interval, which can determine from which level the moderator variables have moderating effects in the relationship between independent variables and dependent variables [68]. Applied to this study, we can measure the moderating effect of social media interaction in the relationship between cultural identity and brand value, so as to obtain the statistical significance interval of the moderating effect. As can be seen from Figure 3, when the standardized value of social media interaction is >4.704, the moderating effect is significantly positive; when the standardized value of social media interaction is <4.704, the moderating effect does not exist. It can be seen that the higher the degree of social media interaction, the more significant the impact of cultural identity on brand value.

## 5. Discussion and Conclusions

This study selects the Anxi Tieguanyin Tea Culture System as a research case, and verifies the interaction relationship between cultural memory, cultural identity, brand value, and social media of agricultural heritage through the research method of the structural equation model. In the past, the research on the brand value of agricultural heritage only stayed in the construction and evaluation of the value system, and the concept of cultural memory has not been introduced into the research of agricultural heritage. Therefore, this study uses quantitative research methods, according to the unique cultural attributes of agricultural heritage, discusses the impact of cultural memory of agricultural heritage on brand value for the first time, supplements and enriches the research perspective of agricultural heritage, and puts forward some suggestions for management practice.

Firstly, cultural memory has a significant positive impact on brand value. The richer the cultural memory of agricultural heritage, the deeper the cultural attributes injected into brand value, and the higher the degree of recognition of brand value. This result echoes the views put forward by Unurlu [13] and Um, Dong [15]. Cultural identity plays a partial mediation role in the impact of cultural memory on brand value. Cultural memory not only directly affects brand value (84.3%), but also partly affects brand value (15.7%) through cultural identity. In other words, the impact of cultural memory on brand value is divided into direct and indirect impact paths. On the one hand, it directly affects the promotion of brand value; on the other hand, cultural memory will make the group produce cultural identity, thus affecting the group’s cognition of brand value. The recognition of agricultural heritage by academia and business circles, the support of relevant government policies for agricultural heritage, and the recognition of agricultural heritage by relevant international measures all promote the group to accept the content of agricultural heritage and form cultural identity, which is consistent with the view of Yucel [7]. Group acceptance deepens with the occurrence of the above acts of authoritative organizations, thereby recognizing the brand’s product quality, production process, planting ecology, and cultural identity to improve consumers’ preferences and purchasing power for local brands [8], so as to enhance the brand value of agricultural heritage.

Secondly, social media interaction moderates the relationship between cultural identity and brand value. The higher the degree of social media interaction, the greater the impact of cultural identity on brand value. Different from mass media, social media has the characteristics of simplicity, immediacy, and interactivity [38]. The use of social media regulates the impact of network diversity on the group’s cultural knowledge [11]. Communicators can promote the group’s cultural identity of agricultural heritage through social media. Effective communication will increase the group’s cultural identity of agricultural heritage, give the group a sense of interest and trust in it, and improve the brand value. It should be noted that only when the standardized value of social media interaction is >4.704, the moderating effect can be significant. It shows that in the context of the Internet, the more frequently the group communicates on social media, the more significant the role of their cultural identity in promoting brand value.

Thirdly, the impact of social media interaction on cultural memory and brand value, and the impact of social media interaction on cultural memory and cultural identity, have not reached a significant level. This is not completely consistent with the previous research results. The possible reason is that Laroche, Khamis, Mathur, and other research objects are active communicators in social media, who can actively guide groups to discuss product culture, so that the influence of social media interaction is obvious [33,44,48]. However, the agricultural heritage discussed in this study has not yet been a topic of agricultural heritage manufacturing by communicators, and the network environment has not yet triggered a hot discussion of agricultural heritage by groups. Although a large amount of content about agricultural heritage has emerged in social media, the group has less discussion on agricultural heritage on the Internet. Therefore, social media interaction has not affected the relationship between the cultural memory and cultural identity of agricultural heritage, nor has it directly affected the relationship between cultural memory and brand value.

### 5.1. Theoretical Implications

Firstly, this study defines the core concept of cultural memory of agricultural heritage for the first time by using the grounded theory method, carries out empirical analysis, uses a structural equation model to estimate the importance of each dimension of cultural memory of agricultural heritage, and simplifies and improves the scale of cultural memory of agricultural heritage through model modification, providing a reference measurement framework for subsequent research.

Secondly, this study uses grounded theory to analyze the influence mechanism of cultural memory on the brand value of agricultural heritage, and carries out an empirical test, which expands the research framework of agricultural heritage, introduces mixed research methods into the research field of agricultural heritage, and promotes its theoretical research. At present, most studies on the value of agricultural heritage are value evaluation, and this study explores the factors that influence the promotion of brand value in the value of agricultural heritage through empirical analysis, so as to provide reference for the countermeasures of brand value promotion. This paper adopts mixed research methods, uses the grounded theory analysis method to construct a theoretical dimension of cultural memory of agricultural heritage, and uses the statistical analysis method to explore the composing dimension of cultural memory of agricultural heritage and its influence mechanism on brand value, so as to put forward suggestions on the promotion of brand value of agricultural heritage.

### 5.2. Managerial Implications

According to the research results, the promotion of the brand value of agricultural heritage should be carried out from the following aspects: firstly, to improve the popularization rate of cultural memory of agricultural heritage; secondly, to pay attention to the group’s cultural identity of cultural memory; thirdly, to use social media interaction to guide group cultural identity, so as to enhance the brand value of agricultural heritage.

In terms of improving the popularity of cultural memory of agricultural heritage, we should give full play to the communication power of government, enterprises, universities, and other authoritative institutions, and positively guide the communication of cultural memory. On the one hand, the disseminators spread cultural memory to the group through the media after screening and checking the content; on the other hand, the group feeds back their views on cultural memory to the disseminators, who optimize the form and content of communication. The correct communication mode to improve the group’s awareness of agricultural heritage and make the cultural memory of agricultural heritage accepted by a wider group is the key basic link to enhance the brand value of agricultural heritage.

In terms of strengthening the group’s cultural identity to cultural memory, it is important to give full play to the influence of authoritative institutions and guide the group to produce cultural identity to cultural memory. Authorities should do a good job in the promotion and publicity of cultural memory, correctly guide some heritage groups that first contact cultural memory, promote the dissemination of cultural memory by communicators, and accept and integrate other groups that lag in contact with cultural memory into the ranks of cultural memory carriers, thereby promoting the group’s cultural identity to cultural memory. In this process, communicators should adjust the publicity methods and efforts at any time according to the recognition effect of the group on cultural memory, so as to achieve better cultural recognition and enhance the promotion of brand value.

In terms of using social media interaction, authoritative institutions should create hot topics of agricultural heritage in real time to improve the interaction activity between communicators and groups. It is necessary to pay attention to the moderating role of social media in the process of cultural memory communication and feedback. The official media should control the communication direction, pay attention to the formation of opinion leaders in the communication process, and guide the dissemination and exchange of positive information of cultural memory, thereby making the group have cultural identity and enhancing the brand value of agricultural heritage.

### 5.3. Limitations and Future Study

Due to various subjective and objective reasons, this study has some limitations. Firstly, there is no mature scale for the cultural memory of agricultural heritage, so this study uses the grounded theory method to develop the scale. Although this study adopts the method of team discussion to reduce the subjectivity in the process of grounded theoretical analysis, and adopts the Delphi method, it cannot completely eliminate the subjectivity of qualitative research, and the accuracy of the theory needs to be verified repeatedly. Secondly, although the survey sample size of this study meets the basic quantity requirements of factor analysis, the sample size is still not rich due to the limitations of time and resources, which limits the representativeness of samples and the applicability of research conclusions to a certain extent. Therefore, future research can collect more diverse and representative samples in a larger range to further verify the applicability of the scale. Finally, although this study takes the Anxi Tieguanyin Tea Culture System as a case to discuss the impact of cultural memory of agricultural heritage on brand value, which is widely representative, considering the differences between cultural memory of agricultural heritage and brand value in different regions, in the future, the process model should be improved and verified according to the actual application scenarios to obtain more accurate research results.

## Figures and Tables

**Table 1:** Variables and codes.

Constructs	Items	Item Descriptions	Scale Source
Physical Cultural Memory	SC1	I know Anxi Tieguanyin Tea	Extraction of Grounded Theory Codes
	SC2	I know the tea set for brewing Anxi Tieguanyin Tea	
	SC3	I know the farm tools for making Anxi Tieguanyin Tea	
Spiritual Cultural Memory	MC1	I know the tea ceremony spirit of Anxi Tieguanyin Tea	
	MC2	I have heard of the mythical origin of Anxi Tieguanyin Tea	
	MC3	I have heard of religious beliefs related to Anxi Tieguanyin Tea	
Behavioral Cultural Memory	OC1	I know Anxi Tieguanyin Tea Contests or Tea King Competition and other tea customs	
	OC2	I know the sales channel of Anxi Tieguanyin Tea products	
	OC3	I know something about Anxi Tieguanyin Tea culture tourism	
Institutional Cultural Memory	IC1	I know Anxi Tieguanyin Tea is a China Nationally Important Agricultural Heritage System (China-NIAHS)	
	IC2	I have heard of the trade of Anxi Tieguanyin Tea on the Silk Road	
	IC3	I know some cooperatives or organizations involved in the Anxi Tieguanyin tea industry	
Belonging Layer Cultural Memory	CC1	I know about oolong tea culture	
	CC2	I know something about Chinese tea culture	
	CC3	I paid attention to Anxi Tieguanyin Tea culture by contacting oolong tea culture and Chinese tea culture	
Similar Layer Cultural Memory	LC1	I am interested in wine culture	
	LC2	I am interested in coffee culture	
	LC3	I am interested in milk tea	
	LC4	I am interested in wine culture, coffee culture, or milk tea, and further pay attention to the Anxi Tieguanyin Tea Culture System	
Farming Civilization Layer Cultural Memory	AC1	I know of farming civilization	
	AC2	I know of agricultural heritage	
	AC3	I am interested in farming civilization and agricultural heritage, so I came into contact with Anxi Tieguanyin Tea culture	
Space–time Layer Cultural Memory	TSC1	I know something about the culture and history of Anxi County	
	TSC2	I know some cultural history of Quanzhou	
	TSC3	I know Fujian Province is a big tea producing province	
	TSC4	I paid attention to the Anxi Tieguanyin Tea Culture System by understanding the culture of Anxi County, Quanzhou city, or Fujian Province	
Cultural Identity	CI1	Anxi Tieguanyin Tea Culture System is an important part of my understanding of tea culture	Peng [62]
	CI2	Anxi Tieguanyin Tea Culture System is important to me	
	CI3	I am proud of the Anxi Tieguanyin Tea Culture System and its honors at home and abroad	
	CI4	I am very interested in the Anxi Tieguanyin Tea Culture System	
	CI5	When Anxi Tieguanyin Tea Culture System was recognized by others, I felt as if I had made some achievements	
	CI6	I will discuss the Anxi Tieguanyin Tea Culture System with people with similar cultural backgrounds	
Brand Value	BV1	Anxi County has been identified as an important agricultural heritage site, and the Anxi Tieguanyin Tea Culture System as an agricultural heritage, so its brand guarantees us the high quality of tea	Ghosh [63] Kim [64]
	BV2	Anxi County has been identified as an important agricultural heritage site, and the Anxi Tieguanyin Tea Culture System as an agricultural heritage, so its brand ensures that the production process of tea is traditional and standardized	
	BV3	Anxi County has been identified as an important agricultural heritage site, and the Anxi Tieguanyin Tea Culture System as an agricultural heritage, so its brand ensures that the ecology of tea plantations meets the standard	
	BV4	Anxi County has been identified as an important agricultural heritage site, and the Anxi Tieguanyin Tea Culture System as an agricultural heritage, so its brand is committed to improving the evaluation of consumers	
	BV5	It is an important goal of the brand to distinguish from the types of tea in the same field	
Social Media Interaction	WI1	I will get information about the Anxi Tieguanyin Tea Culture System from the Internet platform	Albert [40]
	WI2	I will participate in the discussion on Anxi Tieguanyin Tea culture or brand on the Internet platform	
	WI3	I will change my views on Anxi Tieguanyin Tea culture or brand according to the comments in the comment area	
	WI4	I will buy Anxi Tieguanyin Tea products on the Internet platform	
	WI5	I can get feedback on the evaluation of Anxi Tieguanyin Tea products on the Internet platform	

**Table 2:** Demographic profile of the sample.

Demographic	Frequency	Percentage
**Gender**		
Male	297	51
Female	285	49
**Age**		
18–25	175	30.1
26–35	168	28.9
36–45	135	23.2
46–60	76	13.1
>60	28	4.8
**Education**		
High school and below	72	12.4
Junior college	142	24.4
Undergraduate	278	47.8
Graduate and above	90	15.5
**Monthly Income (RMB)**		
2000<	22	3.8
2000–4999	173	29.7
5000–7999	181	31.1
8000–10,000	119	20.4
>10,000	87	14.9
**Occupation**		
Anxi Tieguanyin grower or tea enterprise practitioner	119	20.4
Enterprise practitioners (non-tea enterprises)	151	25.9
Government personnel	117	20.1
College teachers or researchers	122	21
Media practitioners	41	7
Student	27	4.6
Other	5	0.9
**Origin**		
Within the heritage site	333	57.2
Outside the heritage site	249	42.8

**Table 3:** Exploratory factor analysis.

Constructs	Items	Factor 1	Factor 2	Factor 3	Factor 4	Factor 5	Factor 6	Factor 7	Factor 8	Factor 9	Factor 10	Factor 11
Cultural Identity	CI1	0.870										
	CI2	0.849										
	CI3	0.814										
	CI4	0.806										
	CI5	0.773										
	CI6	0.770										
Brand Value	BV1		0.854									
	BV2		0.851									
	BV3		0.841									
	BV4		0.800									
	BV5		0.784									
Social Media Interaction	WI1			0.856								
	WI2			0.853								
	WI3			0.818								
	WI4			0.798								
	WI5			0.784								
Similar Layer Cultural Memory	LC1				0.886							
	LC2				0.869							
	LC3				0.813							
	LC4				0.795							
Space–time Layer Cultural Memory	TSC1					0.874						
	TSC2					0.842						
	TSC3					0.783						
	TSC4					0.780						
Belonging Layer Cultural Memory	CC1						0.928					
	CC2						0.907					
	CC3						0.880					
Farming Civilization Layer Cultural Memory	AC1							0.934				
	AC2							0.903				
	AC3							0.895				
Spiritual Cultural Memory	MC1								0.872			
	MC2								0.828			
	MC3								0.787			
Behavioral Cultural Memory	OC1									0.866		
	OC2									0.859		
	OC3									0.767		
Institutional Cultural Memory	IC1										0.844	
	IC2										0.838	
	IC3										0.793	
Physical Cultural Memory	SC1											0.860
	SC2											0.842
	SC3											0.731

**Table 4:** Reliability and convergence test.

Constructs/Items	Factor Loadings	Cronbach’s α	CR	AVE
**Physical Cultural Memory**				
SC1	0.650	0.786	0.809	0.590
SC2	0.909			
SC3	0.722			
**Spiritual Cultural Memory**				
MC1	0.716	0.812	0.817	0.601
MC2	0.887			
MC3	0.710			
**Behavioral Cultural Memory**				
OC1	0.699	0.805	0.814	0.595
OC2	0.846			
OC3	0.762			
**Institutional Cultural Memory**				
IC1	0.732	0.795	0.799	0.57
IC2	0.772			
IC3	0.760			
**Belonging Layer Cultural Memory**				
CC1	0.861	0.907	0.908	0.767
CC2	0.924			
CC3	0.841			
**Similar Layer Cultural Memory**				
LC1	0.753	0.866	0.867	0.622
LC2	0.842			
LC3	0.844			
LC4	0.706			
**Farming Civilization Layer Cultural Memory**				
AC1	0.843	0.904	0.905	0.762
AC2	0.931			
AC3	0.841			
**Space–time Layer Cultural Memory**				
TSC1	0.748	0.851	0.853	0.594
TSC2	0.826			
TSC3	0.803			
TSC4	0.699			
**Cultural Identity**				
CI1	0.741	0.903	0.904	0.612
CI2	0.778			
CI3	0.871			
CI4	0.823			
CI5	0.757			
CI6	0.715			
**Brand Value**				
BV1	0.704	0.884	0.884	0.606
BV2	0.708			
BV3	0.782			
BV4	0.847			
BV5	0.838			
**Social Media Interaction**				
WI1	0.715	0.886	0.885	0.609
WI2	0.682			
WI3	0.800			
WI4	0.849			
WI5	0.841			

Remark: CR = Composite Reliability, AVE = Average Variance Extracted.

**Table 5:** The discriminant validity.

Latent Variable	WI	BV	TSC	AC	LC	CC	IC	OC	MC	SC	CI
WI	**0.780**										
BV	0.059	**0.778**									
TSC	0.082	0.068	**0.771**								
AC	−0.021	0.061	0.050	**0.873**							
LC	0.031	−0.06	0.045	−0.07	**0.788**						
CC	0.056	0.111	0.088	0.084	−0.113	**0.755**					
IC	0.100	0.031	0.227	0.129	0.049	0.109	**0.569**				
OC	0.053	0.126	0.151	0.068	0.019	0.124	0.203	**0.772**			
MC	0.137	0.096	0.219	−0.014	0.074	0.139	0.168	0.192	**0.775**		
SC	-0.001	0.107	0.208	0.044	0.112	0.085	0.271	0.213	0.231	**0.768**	
CI	0.036	0.137	0.144	0.045	−0.048	0.172	0.090	0.152	0.181	0.182	**0.782**

Remark: Bold front = square-root of AVE.

**Table 6:** Results of the model fit measures.

Index	χ^2^	df	χ^2^/df	GFI	AGFI	CFI	RMSEA
Initial Model	598.641	149	4.018	0.904	0.877	0.891	0.072
Modified Model	278.403	116	2.400	0.946	0.929	0.948	0.049
Recommended Value	-	-	<3	>0.9	>0.9	>0.9	<0.05

**Table 7:** Hypothesis test results.

Variable	Equation (1) (Dependent Variable: Brand Value)			Equation (2) (Dependent Variable: Cultural Identity)			Equation (3) (Dependent Variable: Brand Value)		
	β	se	*t*	β	se	*t*	β	se	*t*
Constant	3.542	0.488	7.263	2.672	0.505	5.289	3.290	0.497	6.616
Gender	−0.028	0.095	−0.299	−0.123	0.098	−1.254	−0.017	0.094	−0.177
Age	0.104	0.040	2.574	0.081	0.042	1.941	0.096	0.040	2.386
Education	−0.019	0.054	−0.352	0.019	0.056	0.347	−0.021	0.054	−0.387
Income	−0.003	0.043	−0.068	0.039	0.045	0.877	−0.007	0.043	−0.154
Cultural Memory	0.254	0.078	3.258	0.425	0.081	5.263	0.214	0.080	2.692
Cultural Identity							0.094	0.040	2.348
R-sq	0.034			0.065			0.043		
F Value	4.002			8.030			4.280		

**Table 8:** Breakdown of total effect, direct effect, and mediating effect.

Project	Effect	Boot Standard Error	Boot CI Lower Limit	Boot CI Upper Limit	Relative Effect Value
Total Effect	0.254	0.084	0.089	0.415	
Direct Effect	0.214	0.084	0.053	0.384	84.3%
Mediating Effect	0.040	0.020	0.004	0.082	15.7%

**Table 9:** Moderated mediation model test.

Variable	Equation (4) (Dependent Variable: Cultural Identity)			Equation (5) (Dependent Variable: Brand Value)		
	β	se	*t*	β	se	*t*
Constant	−0.244	0.293	−0.833	4.790	0.281	17.033
Gender	−0.120	0.098	−1.214	−0.000	0.094	−0.003
Age	0.080	0.042	1.914	0.093	0.040	2.314
Education	0.013	0.056	0.237	−0.008	0.054	−0.140
Income	0.040	0.045	0.882	−0.010	0.043	−0.237
Cultural Memory	0.420	0.081	5.159	0.221	0.080	2.776
Cultural Identity				0.033	0.039	0.845
Social Media Interaction	0.009	0.041	0.214	0.094	0.040	2.360
Cultural Memory×Social Media Interaction	−0.055	0.057	−0.959	−0.026	0.059	−0.431
Cultural Identity×Social Media Interaction				0.079	0.033	2.410
R-sq	0.067			0.054		
F value	5.860			3.635		

**Table 10:** Bootstrap test with moderated mediator effect.

Result Type	Moderator Variable	Effect	Boot SE	Boot95% CI	
				Low	High
Moderated Mediator Effect	Low Social Media Interaction-Eff1(M-1SD)	−0.001	0.030	−0.064	0.058
	Medium Social Media Interaction-Eff2(M)	0.040	0.020	0.004	0.081
	High Social Media Interaction-Eff3(M + 1SD)	0.067	0.031	0.016	0.138
Moderated Mediator Effect Comparison	(Medium–Low) Eff2-Eff1	0.040	0.022	0.000	0.086
	(High–Low) Eff3-Eff1	0.068	0.043	−0.009	0.160
	(High–Medium) Eff3-Eff2	0.028	0.026	−0.014	0.086

## Data Availability

Data are available in the supplementary article file.

## Supplementary Materials

The following supporting information can be downloaded at: https://www.mdpi.com/article/10.3390/bs13020079/s1, Table S1: Inter-item covariance matrix.
